# Assessment of Cognitive Symptoms in Prodromal and Early Huntington Disease

**DOI:** 10.1371/currents.RRN1250

**Published:** 2011-10-25

**Authors:** Anthony L Vaccarino, Terrence Sills, Karen E. Anderson, Beth Borowsky, David Craufurd, Joseph Giuliano, LaVonne Goodman, Mark Guttman, Peter Kupchak, Aileen K Ho, Jane S. Paulsen, Julie C. Stout, Daniel P van Kammen, Ken Evans

**Affiliations:** ^*^Research Methods, Ontario Cancer Biomarker Network, Toronto, Ontario, Canada; ^‡^Department of Psychiatry and Department of Neurology, University of Maryland, School of Medicine, Baltimore, MD USA; ^§^Translational Medicine, CHDI Foundation, Inc., Princeton NJ; ^¶^University of Manchester, Manchester Academic Health Sciences Centre and Central Manchester University Hospitals NHS Foundation Trust, Manchester, UK; ^#^CHDI Foundation, Inc.; ^**^Huntington's Disease Drug Works, Lake Forest Park, WA; ^††^Division of Neurology, Department of Medicine, University of Toronto, Toronto, Ontario Canada; ^§§^School of Psychology and Clinical Language Sciences, University of Reading, U.K.; ^¶¶^Department of Psychiatry, The University of Iowa Carver College of Medicine, Iowa City, IA, USA; ^##^School of Psychology and Psychiatry, Monash University, Melbourne, Australia and ^***^CNS Drug Development consultant

## Abstract

The Functional Rating Scale Taskforce for pre-Huntington Disease (FuRST-pHD) is a multinational, multidisciplinary initiative with the goal of developing a data-driven, comprehensive, psychometrically sound, rating scale for assessing symptoms and functional ability in prodromal and early Huntington disease (HD) gene expansion carriers. The process involves input from numerous sources to identify relevant symptom domains, including HD individuals, caregivers, and experts from a variety of fields, as well as knowledge gained from the analysis of data from ongoing large-scale studies in HD using existing clinical scales. This is an iterative process in which an ongoing series of field tests in prodromal (prHD) and early HD individuals provides the team with data on which to make decisions regarding which questions should undergo further development or testing and which should be excluded. We report here the development and assessment of the first iteration of interview questions aimed to assess cognitive symptoms in prHD and early HD individuals.

## Introduction

Earliest clinical manifestations of Huntington disease (HD) are poorly characterized, and there is a need for clinical scales specifically designed to measure early changes in HD gene expansion carriers. The Functional Rating Scale Taskforce for pre-Huntington Disease (FuRST-pHD) is a multinational, multidisciplinary collaboration to develop a valid functional rating scale to assess changes in symptom severity in HD gene expansion carriers who do not yet meet criteria for a formal clinical diagnosis (prodromal HD or prHD) or are early manifest.[1] Such a measurement tool is essential to better understand the earliest manifestations of HD, and to evaluate novel therapies early in the course of disease. 

FuRST-pHD has established an inclusive process using input from numerous sources, including prHD and early HD individuals, caregivers, and experts from a variety of fields, as well as from ongoing large-scale HD studies using existing clinical scales.[1] As part of the process, an inclusive series of “Working Groups” of individuals with clinical and/or scale development expertise have been established to review existing data and develop interview questions within the specific domain under study. Once these interview questions are developed, they are distributed to trained raters for beta testing in gene expansion carriers. This is an iterative process, in which changes or deletions (as appropriate) are made based on empirical evidence obtained during field testing; the modified questions are then tested during subsequent iterations so that the list can ultimately be winnowed to select optimal items for scale inclusion. 

Cognitive-related impairments and decline are thought to be a prevalent component of HD and present in prHD.[2] However, these symptoms are incompletely understood, and the presence of other manifestations (i.e., psychiatric and motor) may confound assessment. Furthermore, although cognitive batteries used to assess cognitive function show impairment in prHD,[2] the degree that these changes are clinically meaningful has not been determined. Indeed, it is the position of the FDA that in addition to cognitive batteries to assess changes in cognitive performance, co-primary measures should also assess a clinically-meaningful functional outcome, including interview-based measures of cognition.[3] As such, current data would benefit from evidence indicating that these changes are associated with symptoms that can be perceived by the patient (and therefore also the clinician) and interfere with their day-to-day functioning; in developing interview questions the working group considered the possible functional correlates of those changes. We report here the development and assessment of interview questions aimed to assess cognitive-related symptoms in prHD/early HD individuals.

## Methods 

A two-day Cognitive Symptoms Working Group meeting was held in Toronto, Canada, October 1 and 2, 2009. The working group charge was to review available evidence and develop interview questions to assess cognitive-related symptoms in prHD.  

### Evidence Reviewed

Data Mining. Although existing tools were not specifically designed to assess early manifestations in HD gene carriers, studies using such measures can nevertheless provide rich and useful information about the expression of symptoms in the target population, the differentiation of early changes from those expressed in advanced disease, or similar symptoms seen in other disorders. There are a number of ongoing studies investigating the symptomatology and progression of prHD that are accessible to the FuRST-pHD program, including PREDICT-HD. To this end, data from PREDICT-HD assessing cognitive-related symptomtology in prHD were reviewed and considered by the working group in developing the interview questions, including the UHDRS Cognitive Subscale, as well as cognitive-related items from the Frontal Systems Behavior Scale, Symptoms Checklist-90, and Beck Depression Inventory.

Patient and Companion Input. The FDA views input from participants, caregivers, and family members as an essential element in developing valid clinical assessment tools.[4] To ensure that the scale reflects concepts that are important from the participant's perspective, patient/companion focus groups were held to identify early symptoms experienced by HD gene expansion carriers. The focus groups were held in a number of countries using the local languages (France, Netherlands, United Kingdom, United States, Portugal, and Spain) with all participants (prHD, early HD and companions) being asked a series of open-ended questions related to symptom occurrence in prHD. All focus group sites had IRB/EC approval and all participants provided informed consent. These data were used to maximise symptom assessment and considered in developing the interview questions.

Cognitive-related symptoms were reported as frequently occurring in prHD and reported to interfere with day-to-day function (Figure 1). 

**Figure:**
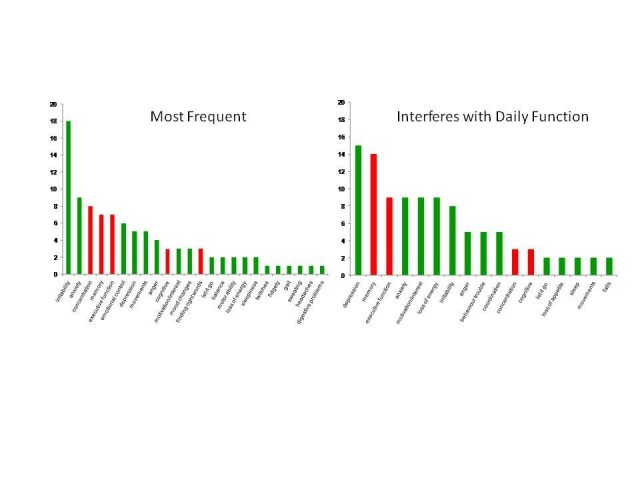
**Figure 1**. Client/caregiver focus groups: Symptom frequency and impact  (French, Dutch, USA, Spanish, Portuguese; number reporting)

The following cognitive-related changes were reported by the focus groups as present during prHD:

**Figure:**
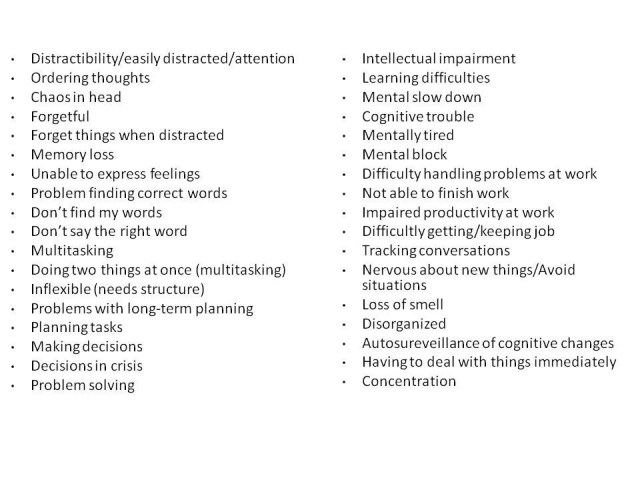

Expert Opinion and Experience of Participants. In addition to reviewing existing data, published literature and working group participant experiences and opinion were also discussed, both within HD and in other disorders.

### Development of Interview Questions 

Based on data mining, the gene expansion carrier, caregiver, expert, and literature input symptom domains and definitions were identified that are thought to be important to prHD. These diverse sources of information provided an excellent starting point for establishing which changes are important to participants. After review of existing data, 11 interview questions were developed to assess specific cognitive-related symptoms and determine their severity (Table 1). 

**Table 1.** Interview questions

**Table:** 

**Interview Question**	**Description/definition**
**Concentration**	Assesses ability to direct and maintain focus on specific activities.
**Odor detection**	Assesses ability to detect odors.
**Social cognition^a^**	Assesses ability to recognize what other people are feeling or intending in his/her interaction with them.
**Interpersonal awkwardness** **^a^**	Assesses ability to appropriately respond to others in social interaction. Does not assess social anxiety.
**Multitasking ** **(concurrent tasks)**	Assesses ability to perform multiple tasks concurrently.
**Multitasking (switching)**	Assesses ability to switch from one task to the next and back again.
**Planning**	Assesses ability to successfully plan and execute plans.
**Prospective memory** **^a^**	Assesses remembering to perform a planned action or intention at the appropriate time.
**Keeping track of time**	Assesses ability to judge how long tasks may take and to sense passage of time.
**Problem solving** **^a^**	Assesses ability to solve problems in day to day activities.
**Expressing thoughts verbally** **^a^**	Assesses ability to express their words or thoughts.

^a^Assessed as symptom frequency only

FuRST-pHD has adopted a semi-structured clinician-administered interview similar to that used for the GRID-HAMD. The GRID format directs the rater to score symptom frequency and intensity separately, while giving them clear scoring anchors, overall definitions, and a semi-structured interview guide with questions to establish symptom experience and determine the specific rating. A composite severity score is derived that corresponds to the appropriate symptom frequency and intensity. This method has been employed successfully and is user-friendly, with acceptable agreement among independent raters.[5] The working group developed interview questions, including structured interview guides, scoring conventions, scoring anchors and symptom definitions. Following the meeting, draft interview questions were circulated for comment on a shared internet site (Sharepoint).

### Field Testing of Interview Questions

Field testing of interview questions in prHD (UHDRS Diagnostic Confidence Level < 4) and early HD (within 5 years from onset of clinical motor signs) was conducted at independently contracted sites. All data collection sites had IRB/EC approval and all participants provided informed consent. Prior to conducting the clinical interview, all raters were trained (via webinar or in person) to ensure that all trainees had an adequate conceptual understanding for administering and scoring each of the items. A minimum sample size of 100 was targeted.

### Data Analysis 

The distribution of the composite score for each individual item was compiled, and summary statistics associated with each item score were computed. Distributions of item scores for prHD and HD subgroups were statistically compared using the non-parametric Mann-Whitney U test.  

Non-parametric item response analyses were performed to determine the relationship between scores on the individual interview questions and total score. Item Response Theory has been demonstrated to be useful in evaluating the performance of individual items (symptoms) on rating scales, by assessing the relationship between a score assigned to an item and the overall severity of the disease[6] [7]. In order to apply IRT to early scale development work we utilize the non-parametric TESTGRAF software and IRT models[6] to generate Option Characteristic Curves (OCCs) that display the probability of a particular option score (i.e., a score of 0, 1, 2, 3, 4) on each interview question as a function of overall level of severity. In the present analyses, total item score was used as a measure of severity. To illustrate this, Figure 2 depicts a hypothetically ‘‘ideal’’ item from an item response perspective, which is characterized by a clear identification of the range of severity scores over which an option is most likely to be endorsed, rapid changes in the curves that correspond to changes in severity, and an orderly relationship between the weight assigned to the option and the region of severity over which an item is likely to be endorsed. As such, OCCs provide a graphical representation of how informative a particular item (or symptom) is as a measure of illness. Frequency distribution of option scoring within each interview question were also generated.

**Figure:**
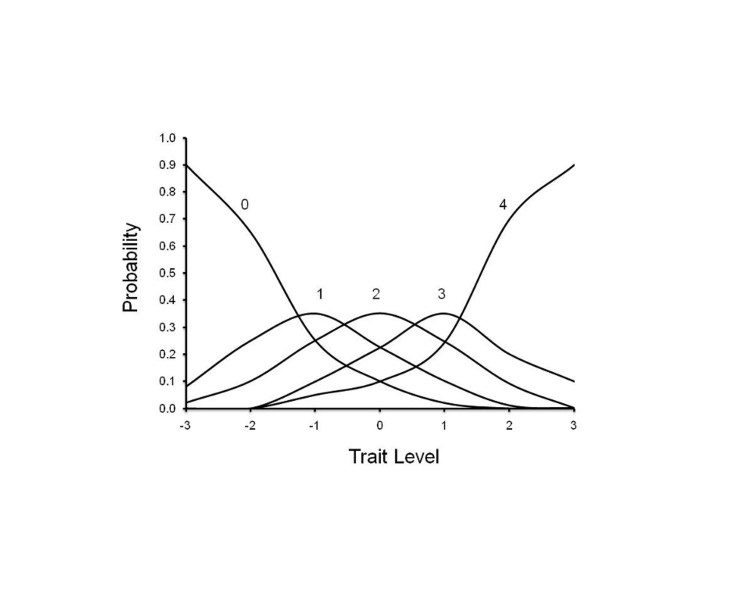
**Figure 2**. OCC for a hypothetically ‘‘ideal’’ item. The OCC shows the probability (*y*-axis) of endorsing a particular option for the item at different levels of the trait (*x*-axis). 

Interview questions which were found to produce scoring and discrimination across ranges of overall severity were selected for further testing. Total subscale scores for prHD and HD subjects were computed and compared statistically using the Mann-Whitney U test. The measure of internal consistency between selected items was estimated using Cronbach's alpha, and the corrected item-total correlation (between each individual item score and the total of the other selected items) was computed. Correlations between the total score and scores of individual questions not selected for further testing were also computed.

## Results

A total of 100 CRFs were completed. The participant demographic characteristics are shown in Table 2.

**Table 2**. Demographic Characteristics

**Table:** 

** **	** All Subjects **	** prHD**	** HD**
**Sample size**	N=100	N=66 (66%)	N=34 (34%)
**Male gender**	N=38 (38%)	N=19 (29%)	N=19 (56%)
**Age**	46.2 (18-79)	43.0 (18-70)	52.2 (35-79)

A follow-up meeting (via webinar) with the working groups was held to review data and make recommendations in moving forward, including item deletion and modification/refinement. The FDA PRO Guidance was used to guide the decision making process.[4] 

OCCs and scoring frequency distributions were generated for each of the interview questions. Of the 11 tested, 5 interview questions were found to produce scoring and discrimination across ranges of severity (Figures 3 and 4, as examples):

### ✓  Concentration

### ✓  Multitasking (Concurrent)

### ✓  Multitasking (Switching)

### ✓  Prospective memory

### ✓  Expressing thoughts verbally

The internal consistency of these five items, as measured by Cronbach's alpha, was 0.80 with respect to the entire study population, 0.85 with respect to the prHD subgroup and 0.73 with respect to the HD subgroup. All corrected item-total correlations were 0.62 or higher with respect to the prHD subgroup; the only item displaying an item-total correlation of less than 0.4 with respect to the HD subgroup was the "Expressing thoughts verbally" item (Table 3).

The mean total composite score with respect to the above 5 items was 3.66 in prHD subjects and 4.93 in HD subjects; the difference in mean scores was not statistically significant (*p* = 0.11, Mann-Whitney U test). The mean "Multitasking - concurrent tasks" composite score was significantly higher in HD than in prHD (*p* = 0.03, Mann-Whitney U test); none of the other items showed significant differences between prHD and HD subgroups. These results are consistent with previous literature demonstrating a decline in cognitive function in prHD and early HD.[2] [8]

It was agreed that these 5 interview questions would be modified accordingly for testing in subsequent iterations; examination of the OCCs provided data on which decisions could be made as to where modifications should be made to improve item performance, including changes in wording and scoring options. For example, Figure 3 shows that for "multitasking" the options with the highest probably of being scored for symptom intensity increased from "0" to "1" (Mild: Requires effort but able to complete tasks accurately) to "4" (Very severe: Needs to stop one task in order to complete the other, needs to focus on only one thing at a time, major errors); options 2 (Moderate: Expends significant effort to complete tasks accurately) and 3 (Severe: Complete tasks with minor errors) were poorly discriminated from one another and never endorsed as the options with the highest probability of being scored, suggesting these options are used interchangeably and thus could be combined into a single option (i.e., able to complete tasks with significant effort) or changed in wording to better discriminate between these levels of symptom intensity. With respect to the frequency of these symptoms, these data showed that when problems in multitasking do occur they were "much of the time/almost always," and tended not to occur "occasionally" (see Figure 3). By contrast, questions assessing "prospective memory" and "expressing thoughts verbally," frequency of symptoms showed an orderly progression from occurring "occasionally" to "much of the time/almost always" (Figure 4).  

**Figure:**
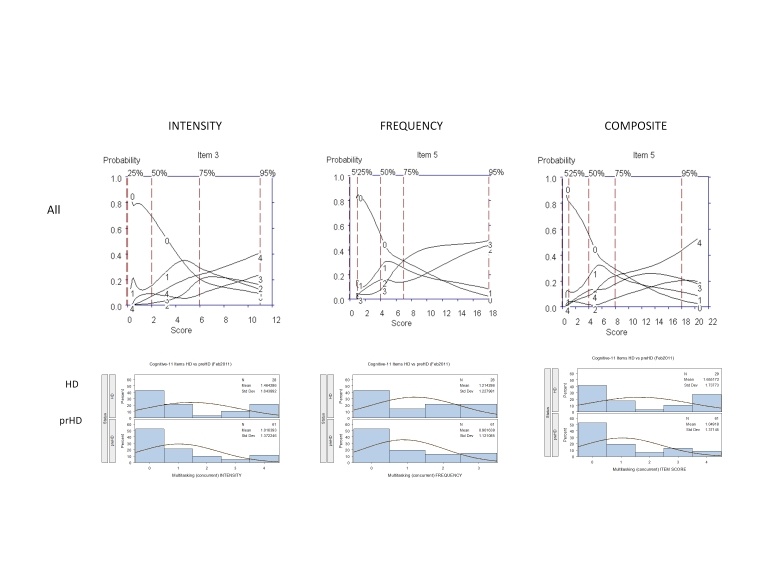
**Figure 3.** OCCs (All participants) and Frequency distribution (HD and prHD) for interview question assessing "Multitasking-Concurrent." Data are presented separately for symptom intensity (left row), frequency (middle row), and composite (right row) scores.

**Figure:**
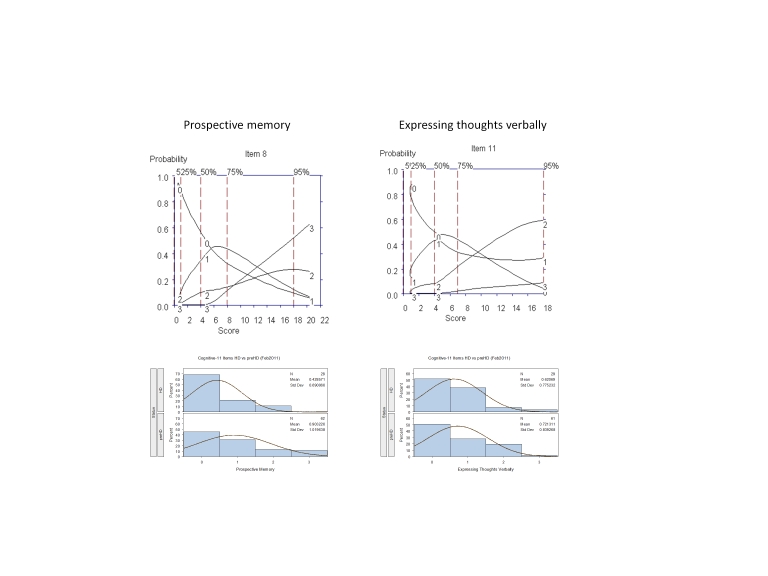
**Figure 4.** OCCs (All participants) and Frequency distribution (HD and prHD) for interview questions assessing frequency of problems with "Prospective memory" and "Expressing thoughts verbally."

**Table 3.  **Correlations between Interview Question Scores

**Table:** 

Item	Item-total correlation (all subjects)	Item-total correlation (prHD subjects)	Item-total correlation (HD subjects)
Concentration	0.585	0.622	0.410
Odor detection	0.245	0.281	0.173
Social cognition	0.484	0.614	0.188
Interpersonal awkwardness	0.514	0.478	0.619
Multitasking (concurrent tasks)	0.655	0.716	0.622
Multitasking (switching)	0.623	0.727	0.464
Planning	0.580	0.563	0.617
Prospective memory	0.640	0.666	0.710
Keeping track of time	0.517	0.557	0.469
Problem solving	0.602	0.717	0.296
Expressing thoughts verbally	0.524	0.616	0.372

The remaining 6 questions were very rarely endorsed and a score of zero had the highest probability of being scored across the full range of severity (Figure 5, as example): 

 ✗ Odor detection ✗ Social cognition ✗ Interpersonal awkwardness ✗ Plannig ✗ Keeping track of time ✗ Problem solving

The mean composite score for these rarely-endorsed items was significantly lower than that for the 5 well-endorsed items (*p* < 0.001, signed rank test). The low frequency of response and poor discriminative properties limit the usefulness of these interview questions for assessment in prHD and early HD. While the correlations between each of these individual item composite scores (with the exception of the "Odor detection" item) and the total score from the 5 highly-endorsed items were generally high with respect to the prHD subgroup (Table 3, unshaded rows), these 6 items were nonetheless rarely endorsed in the general subject population. There were no significant differences in either frequency or severity of symptoms between prHD and HD for any of these 6 items (Figure 5).

**Figure:**
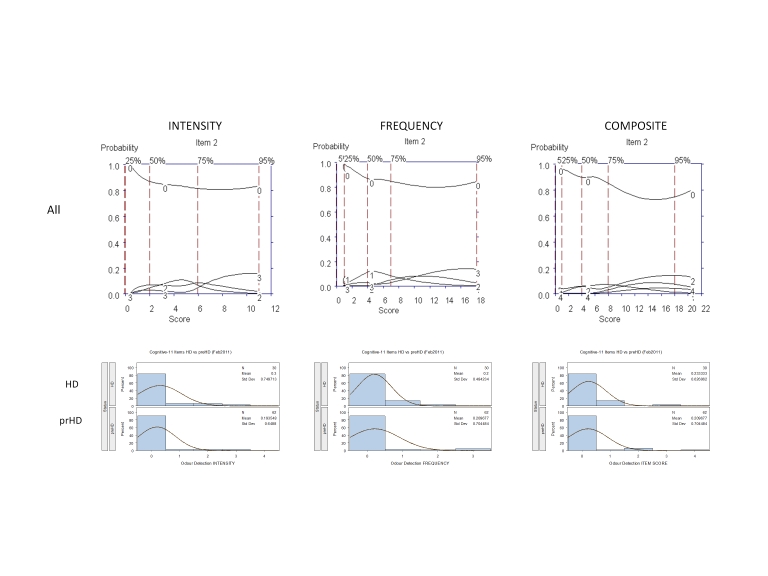
**Figure 5.** OCCs (All participants) and frequency distribution (HD and prHD) of a representative rarely endorsed interview question (Odor detection). Data are presented separately for symptom intensity (left row), frequency (middle row), and composite (right row) scores.

It was agreed that that these 6 interview questions should be removed from subsequent iterations on the basis of Relevance (Reported as not relevant by a large segment of the population of interest) and Response Range (A high percentage of patients respond at the floor) as outlined in Table 1 of the FDA PRO Guidance.[4]

## Discussion

FuRST-pHD has used an inclusive, iterative process to generate interview questions to assess changes in prodromal and early HD gene expansion carriers. Consistent with previous literature,[2] the present results show that many CAG expanded individuals exhibit a range of cognitive-related symptoms prior to clinical diagnosis, although some symptoms are likely to be better candidates for inclusion in a final instrument than others. We report here the development and beta testing of first iteration interview questions designed to assess cognitive-related symptoms. Five interview questions have been selected for further testing, have been modified accordingly by the working groups, and are currently undergoing a second iteration of field testing. The results of the second iteration will be reported once completed.

## Acknowledgments

CHDI Foundation, Inc. – a not-for-profit research organisation whose mission is to rapidly and collaboratively discover and develop therapies that slow the progression of Huntington’s disease – initiated and sponsored the development of the FuRST-pHD. We thank Jamie Levey for her help coordinating the European focus groups, LaVonne Goodman for her help coordinating the USA focus groups, and Stacie Vik and Barbara McQuaid for administrative assistance.

## Funding Information

FuRST-pHD is funded by CHDI. PREDICT-HD is supported by the National Institutes for Health, National Institute of Neurological Disorders and Stroke (NS40068) and CHDI Foundation, Inc

## Competing Interests

The authors have declared that no competing interests exist.

## FuRST-pHD Core Team

K Anderson, B Borowsky, K Evans, J Giuliano, M. Guttman. A Ho, JS Paulsen, T Sills, A. Vaccarino, D van Kammen

## Cognitive Symptoms Working Group

FuRST-pHD Core Team, D Craufurd, L Goodman, P Harvey, J Stout

## Statistics

S Gilbert-Evans, P Kupchak, T Sills, A Vaccarino

## Contributing Field Testing Sites

Birmingham and Solihull Mental Health, Birmingham, UK (Hugh Rickards, MD, Jenny Crooks, BA, Jan Wright, BA); Center for Movement Disorders, Markham, Ontario, Canada (Mark Guttman, MD, Irita Karmalkar, BA, Alanna Sheinberg, BA, and Adam Singer, BA); University of Melbourne, AU (David Ames, MD, Edmond Chiu, MD, Phyllis Chua, MD, Olga Yastrubetskaya, PhD, Joy Preston, Anita Goh, D.Psych, and Angela Komiti, BS, MA); University of Iowa, Iowa City, IA, USA (Leigh Beglinger, PhD., Thomas Wassink, MD, Patricia Ryan, MSW, MA, Stephen Cross, BA, Mycah Kimble, BA, Stacie Vik, BA); Huntington Disease Drug Works, Seattle, WA, USA (LaVonne Goodman, MD); North York General Hospital, Toronto. Ontario, Canada (Clare Gibbons, MS, Jeanne Kennedy, BScNEd, RN, and Wendy Meschino, MD).

## Focus Groups

Portugal (J Ferreira, T Mestre), Spain (A Martínez Descals), France (A Durr, C Jauffret), The Netherlands (R Bos, R Roos, M-N Witjes-Ané), UK (R Fullam, O Handley, J Naji); HD Drug Works, Seattle, USA (L Goodman).

## Corresponding Author

Anthony L Vaccarino, avaccarino@ocbn.ca
